# Phosphorus-acquisition strategies of canola, wheat and barley in soil amended with sewage sludges

**DOI:** 10.1038/s41598-019-51204-x

**Published:** 2019-10-16

**Authors:** C. Nobile, D. Houben, E. Michel, S. Firmin, H. Lambers, E. Kandeler, M.-P. Faucon

**Affiliations:** 1 AGHYLE, SFR Condorcet FR CNRS 3417, UniLaSalle, 19 rue Pierre Waguet, 60026 Beauvais, France; 2 School of Biological Sciences and Institute of Agriculture, The University of Western Australia, 35 Stirling Highway, Crawley (Perth), W A 6009 Australia; 3 Institute of Soil Sciences and Land Evaluation, Soil Biology, University of Hohenheim, Emil-Wolff Str., 27, 70599 Stuttgart, Germany

**Keywords:** Plant sciences, Agroecology, Soil microbiology

## Abstract

Crops have different strategies to acquire poorly-available soil phosphorus (P) which are dependent on their architectural, morphological, and physiological root traits, but their capacity to enhance P acquisition varies with the type of fertilizer applied. The objective of this study was to examine how P-acquisition strategies of three main crops are affected by the application of sewage sludges, compared with a mineral P fertilizer. We carried out a 3-months greenhouse pot experiment and compared the response of P-acquisition traits among wheat, barley and canola in a soil amended with three sludges or a mineral P fertilizer. Results showed that the P-acquisition strategy differed among crops. Compared with canola, wheat and barley had a higher specific root length and a greater root carboxylate release and they acquired as much P from sludge as from mineral P. By contrast, canola shoot P content was greater with sludge than with mineral P. This was attributed to a higher root-released acid phosphatase activity which promoted the mineralization of sludge-derived P-organic. This study showed that contrasted P-acquisition strategies of crops allows increased use of renewable P resources by optimizing combinations of crop and the type of P fertilizer applied within the cropping system.

## Introduction

Phosphorus (P) deficiency in soil restricts the productivity of many agroecosystems. Phosphorus fertilization is mainly based on the application of mineral fertilizer derived from mined phosphate rock, a finite resource which is steadily declining^1^. To meet plant P requirements and sustain the growing population and need for food, we urgently need to explore new strategies that can provide available P for plants while limiting the use of mineral P fertilizer^2^. One solution is recycling P from organic wastes that are rich in P, such as municipal sewage sludge, which represents the main resource of recycled P in the world^3,4^. However, like other organic waste, sewage sludge contains a range of P forms with varying availability to plants compared with conventional P fertilizers in which P is predominantly in a soluble form readily available to plants. Phosphorus is present in sewage sludge as a mixture of both organic and inorganic P forms, in different proportions depending on the origin and the treatment process of sludge^5^. The dominant form of P-organic is inositol hexakisphosphate, phytate^6,7^, while inorganic P forms include calcium (Ca) phosphates and amorphous aluminum (Al)- or iron (Fe)-bound P^8^. These forms need to be transformed to readily available P, which may, therefore, delay the sludge’s fertilizer effect compared with mineral P fertilizer^9^. However, poorly-available P forms in soil or organic waste can be mobilized by some plants and microbes in P deficient soils^10,11^.

Plants have evolved several strategies to improve P availability, especially *via* architectural, morphological and physiological root traits^12,13^. These traits can be grouped in three main P-acquisition strategies: (i) a greater exploration of the soil induced by the development of specific root architecture and morphology; (ii) the mobilization of poorly-soluble inorganic P (Pi) and P-organic by the release of roots exudates, such as protons (H^+^) solubilizing Pi bound to Ca in alkaline soil^14^, or carboxylate increasing P desorption from mineral surfaces such as Al and Fe (hydr)oxides^15^; and (iii) the mineralization of P-organic induced by the production of enzymes such as acid phosphatases^16^. For a sustainable and efficient P fertilization, the challenge is to associate the application of organic waste with crops having traits involved in mobilization of poorly-available P forms.

Different P-mobilization and -acquisition strategies in crop species and cultivars have been highlighted, but their efficiency to acquire P from different P forms in substrates or soil remains poorly studied^17,18^. Recent work of Ceulemans *et al*.^19^ showed that some grassland species had preferential P acquisition from a P source over another, either organic or inorganic P, while others showed equal P acquisition of both P sources. These authors also showed that P acquisition of some grassland species was more efficient when multiple P sources were added to the soil, the addition of both organic and inorganic P to the soil inducing a higher shoot P content than the addition of one form only. However, the relationships between crop P-acquisition traits and preferential acquisition of P sources needs to be determined, especially in soil fertilized with various P forms, such as sewage sludge, compared with a mineral P fertilizer. This knowledge would allow increased use of renewable P resources by matching the type of fertilizer applied and the crop or cultivar with an efficient P-acquisition strategy, and in turn crop succession in crop rotations.

The impact of applied fertilizer on crop P content depends on the interactions with soil microbes, playing a key role in crop P acquisition^20^. Organic waste application, by providing organic C^21,22^, and plants, by releasing substrate or signaling molecules^23^, can enhance the abundance and activity of microorganisms, which can improve P acquisition. The colonization of roots by arbuscular mycorrhizal fungi (AMF) can greatly enhance the soil volume explored^24^. Microorganisms can also exudate carboxylate and phosphatases, but while plants produce principally acid phosphatases, microorganisms can produce both acid and alkaline phosphatases, phytases and phosphonate hydrolases^20^. Therefore, the determination of favorable combinations of organic wastes and crop species requires to investigate plant-microbial interactions.

The objective of the present study was to examine P-acquisition strategies in three main crop species of conventional cropping system under application of different sewage sludges compared with a mineral P fertilizer. We carried out a greenhouse pot experiment and compared the response of P-acquisition traits among wheat, barley and canola in a calcareous soil that received three different sludges or mineral P (triple super phosphate). We hypothesized that (i) crop species studied have different P-acquisition strategies, and (ii) the efficiency of each strategy depends on the type of fertilizer applied. Results obtained could thus help to optimize cropping systems and notably crop rotation using organic waste.

## Results

### Soil phosphorus availability

**Figure 1:**
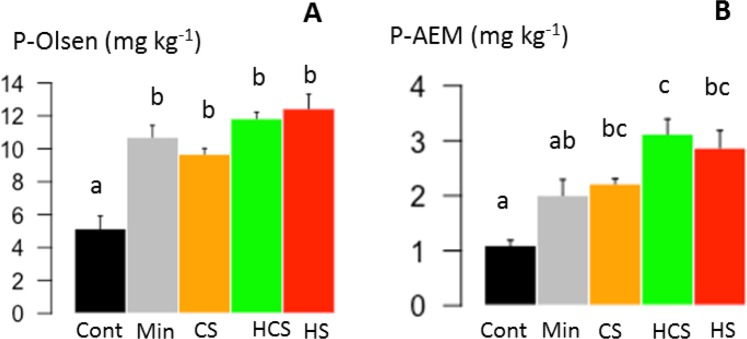
Phosphorus (P) extracted with the Olsen method (P-Olsen) (**A**) or with anion exchange membrane (P-AEM) (**B**) in a calcisol without P fertilization (Cont), fertilized with mineral P (Min), or fertilized with sewage sludge (CS: composted sludge, HCS: heated composted sludge, or HS: heated sludge), after three months without plants, to observe the effect of the fertilization treatment only. Error bars are standard errors (*n* = 4). Different letters indicate significant difference among fertilization treatments at the 0.05 level.

The effect of sludges and mineral P fertilizer application on soil P availability was determined on soils without plants, three months after the application. The application of composted sludge (CS), heated composted sludge (HCS), heated sludge (HS) and mineral P fertilizer (Min) similarly increased the amount of P extracted with the Olsen method (P-Olsen) compared with the control soil (Fig. 1A). The P-Olsen was twofold higher in soils fertilized with sludge (11.3 mg kg^−1^ on average for the three sludges) or mineral P fertilizer (Min: 10.7 mg kg^−1^) than in the control soil (Cont: 5.1 mg kg^−1^). Thus, the application at the same P dose of any of the three sludges had the same effect on soil P-Olsen as the application of mineral P fertilizer.

Amount of P extracted with anion exchange membranes (P-AEM) was two- or threefold higher in sludge-amended soils than in the control soil (Cont: 1.1 mg kg^−1^), while mineral P fertilizer application had no effect on P-AEM compared with the control soil. The HCS (HCS: 3.1 mg kg^−1^) induced a significantly higher P-AEM than the mineral P fertilizer (Min: 2.0 mg kg^−1^) and did not differ from CS and HS treatments (Fig. 1B). Amount of P-Olsen was on average fourfold higher than amount of P-AEM (P-Olsen: 10.0 mg kg^−1^ on average for all treatments; P-AEM: 2.3 mg kg^−1^ on average for all treatments).

### Shoot biomass and phosphorus content

**Figure 2:**
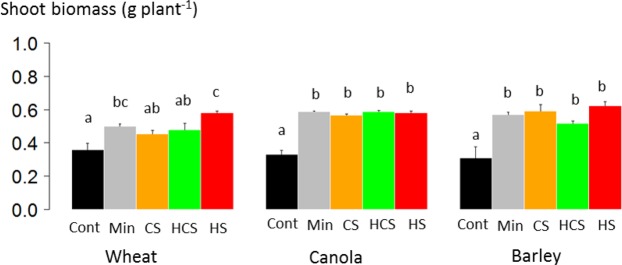
Dry shoot biomass of wheat, canola and barley after three months of growth in a greenhouse on a calcisol without phosphorus (P) fertilization (Cont), fertilized with mineral P (Min), or fertilized with sewage sludge (CS: composted sludge, HCS: heated composted sludge, or HS: heated sludge). Error bars are standard errors (*n* = 4). Different letters indicate significant difference among fertilization treatments at the 0.05 level.

Mineral fertilization as well as sludge addition increased the shoot biomass of canola and barley (Fig. 2). In contrast, the shoot biomass of wheat was only increased by mineral fertilization and heated sludge.

**Figure 3:**
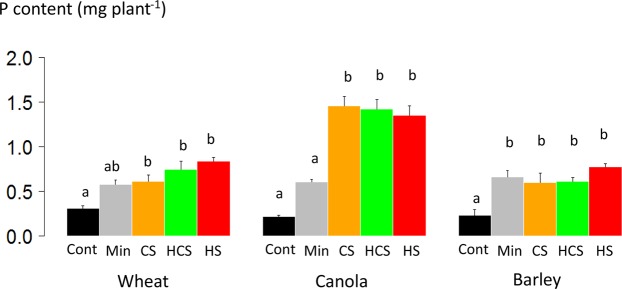
Shoot phosphorus (P) content of wheat, canola and barley after three months of growth in a greenhouse on a calcisol without P fertilization (Cont), fertilized with mineral P (Min), or fertilized with sewage sludge (CS: composted sludge, HCS: heated composted sludge, or HS: heated sludge). Error bars are standard error (*n* = 4). Different letters indicate significant difference among fertilization treatments at the 0.05 level; ns is not significant.

Shoot P content of canola was higher in soils fertilized with any of the three sludges than in soil fertilized with mineral P and in the control soil (Fig. 3). Shoot P content of canola was 2.5 times greater in soils fertilized with sludges (1.4 mg plant^1^, on average for CS, HCS and HS) than in soil fertilized with mineral P (0.6 mg plant^−1^ By contrast, shoot P content of barley was similar in soils fertilized with sludge and mineral P and significantly higher than in the control soil. Barley P content was 0.66 mg plant^−1^, on average, for CS, HCS and HS, and 0.66 mg plant^−1^ for mineral P. Shoot P content of barley was similar in soils fertilized with any of the three sludges and with mineral P but was significantly higher with the sludges than in the control soil. Wheat shoot P content was 0.73 mg plant^−1^, on average, for CS, HCS and HS, and 0.57 mg ind^−1^ for mineral P. Among the three crops, only canola had a significant greater P content in soils fertilized with sludge than from soil fertilized with mineral P.

### Phosphorus-acquisition traits

**Figure 4:**
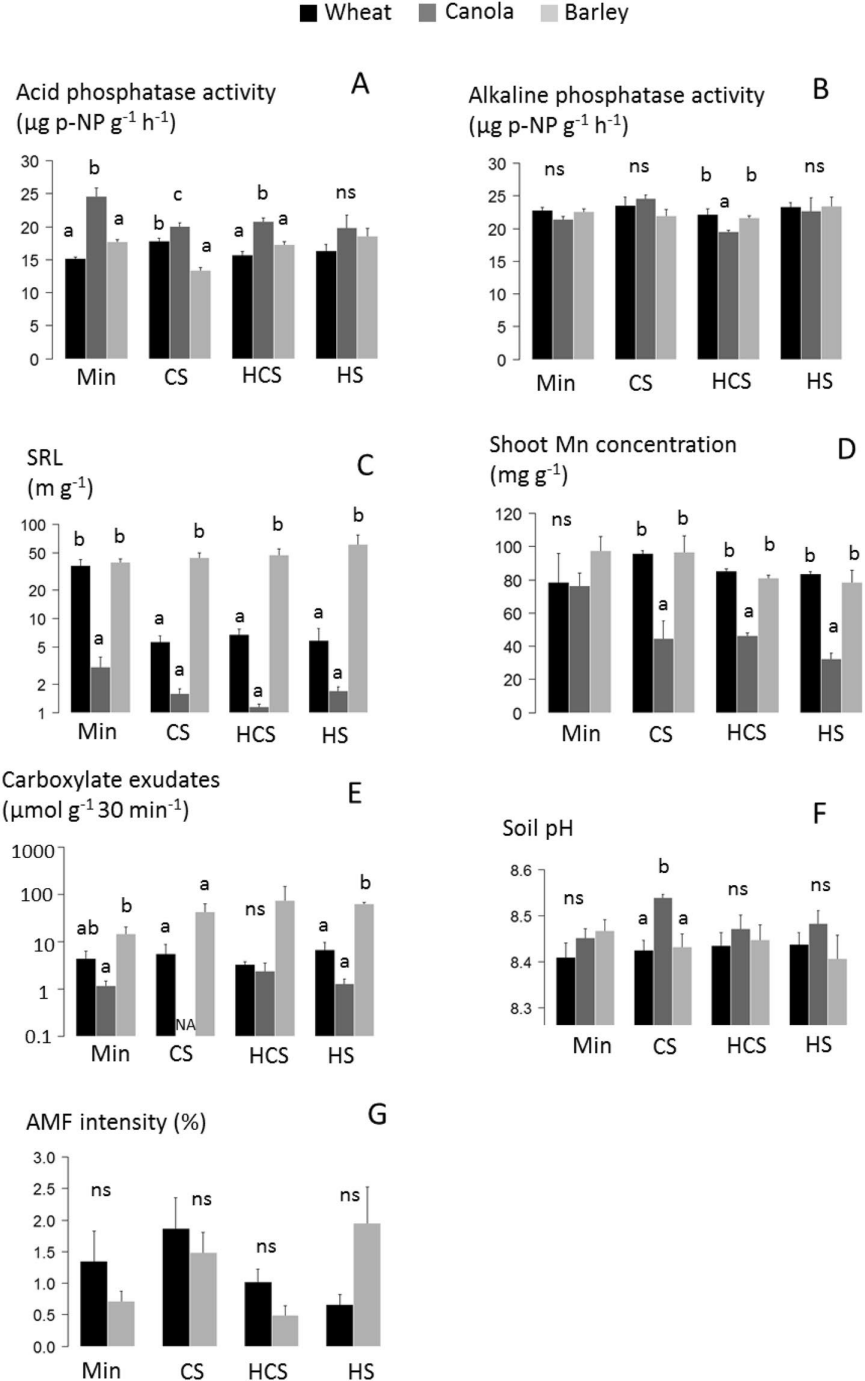
Traits involved in phosphorus (P) acquisition for wheat, canola and barley grown for three months in a calcisol fertilized with mineral P (Min), or fertilized with sewage sludge (CS: composted sludge, HCS: heated composted sludge, or HS: heated sludge). SRL: specific root length; Mn: manganese; AMF: arbuscular mycorrhizal fungi; as Brassicaceae are not colonized by AMF, AMF intensity was presented for wheat and barley only (4E). Error bars are standard errors (*n* = 4). NA is not available. Different letters indicate significant difference among crops at the 0.05 level; ns: no significant difference.

Acid phosphatase activity in the rhizosphere soil was higher for canola than for wheat or barley in soils fertilized with mineral P, CS and HCS (Fig. 4A). The largest differences of acid phosphatase activity between canola and the two other crops were observed in the soil fertilized with mineral P, with 25 µg p-NP g^−1^ h^−1^ for canola, and 15 µg p-NP g^−1^ h^−1^ for wheat and 18 µg p-NP g^−1^ h^−1^ for barley. In contrast, alkaline phosphatase activity in the rhizosheath was similar for the three crops, except in the soil fertilized with HCS, where the activity was lower for canola than for wheat and barley (Fig. 4B).

Specific root length (SRL) was greater for barley than for wheat and canola in soils fertilized with any of the sludges (Fig. 4C). On average, for the three sludges SRL was 51 m g^−1^ for barley and 6 m g^−1^ for wheat and 2 m g^−1^ for canola. In the soil fertilized with mineral P, SRL was the same for barley (40 m g^−1^) and wheat (36 m g^−1^), and lower for canola (3 m g^−1^).

Shoot canola Mn concentration (a proxy for carboxylate in the rhizosheath) was half of shoot wheat and barley Mn concentration in the soils fertilized with any of the sludges (Fig. 4D). Shoot Mn concentration was 41 mg g^−1^, on average, for the three sludges for canola and 86.5 mg g^−1^, on average, for wheat and barley. Total amounts of root carboxylate changed between species. With HS treatment, barley produced significantly higher carboxylate amount than wheat and canola which produced similar carboxylate amount; with Min treatment, barley produced significantly higher carboxylate amount than canola but similar amount than wheat; while with CS and HCS treatments, species produced similar amount of carboxylate (Fig. 4E). Barley produced much greater amounts of malonate (up to 68.6 µmol g^−1^ 30 min^−1^ with HS) compared with wheat (up to 0.5 µmol g^−1^ 30 min^−1^ with CS) and canola (up to 0.1 µmol g^−1^ 30 min^−1^ with mineral P) (Fig. S1A). In addition, barley showed the greatest release of malate (up to 8.5 µmol g^−1^ 30 min^−1^) (Fig. S1B) and wheat the greatest amount of citrate (up to 5.9 µmol g^−1^ 30 min^−1^) (Fig. S1C), while canola showed the lowest (up to 1.1 µmol g^−1^ 30 min^−1^ for malate and 0.3 µmol g^−1^ 30 min^−1^ for citrate). All plants released very low amounts of fumarate and maleate (Fig. S1D,E).

Rhizosphere pH was similar for the three crops, except in soil fertilized with CS, in which the pH was slightly, but significantly higher for canola (pH: 8.5) than for wheat (pH: 8.4) and barley (pH: 8.4) (Fig. 4F). The intensity of roots colonization by AMF was similar for wheat and barley irrespective of the fertilization treatment (Fig. 4G).

Two-way ANOVA analysis testing the effects of fertilization treatment, type of crop, and their interaction on P-acquisition traits showed that the fertilization treatment impacted only phosphatases activities and shoot Mn concentrations (Table 3). The type of crop impacted acid phosphatase activity, SRL, shoot Mn concentration, pH, and intensity of roots colonization by AMF. The interaction between crop type and fertilization treatment impacted acid phosphatase activity, SRL, shoot Mn concentration, and intensity of roots colonization by AMF.

**Table 1:** Properties and phosphorus fractions in sludges.

	Unit, dry mass	CS	HS	HCS
I_ROC_^a^	%	79.9	80.8	84
pH		7.4	8.4	7.3
C/N^b^		9.5	12.3	9.7
C organic^b^	g kg^−1^	185	251.0	170
N-total^c^	g kg^−1^	19.5	20.5	17.6
N-NH4	g kg^−1^	2.9	3.1	3.08
P-total	g kg^−1^	16.6	50.8	20
Pi-water^d^	mg kg^−1^	0.03	0.1	<1
	%Ptot	0.2	0.2	0.2
P-apatite^e^	mg kg^−1^	12.5	32.2	11.7
	%Ptot	75.3	63.4	58.5
Pi-non-apatite^e^	mg kg^−1^	0.8	9.5	4.0
	%Ptot	4.8	18.7	20.0
P organic^e^	mg kg^−1^	3.3	9.1	4.3
	%Ptot	19.9	17.9	21.5

CS: composted sludge; HS: heated sludge; HCS: heated composted sludge.

^a^Proportion of stable organic matter, XP U 44–162.

^b^EN 15936.

^c^EN 13342.

^d^Inorganic phosphorus extracted with water^44^.

^e^SMT (Standards, Measurements and Testing program) protocol^44^.

**Table 2:** Properties of the calcisol sampled at 0–15 cm and used for the pot experiment.

	Unit	Calcisol
clay	%	56
silt	%	34
sand	%	11
pH-water^a^		8.3
CEC^b^	meq 100 g^−1^	19.2
C org^c^	g kg^−1^	37
Pi-water^d^	mg kg^−1^	<1
P total^e^	mg kg^−1^	810
Fe total^e^	g kg^−1^	14.8
Al total^e^	g kg^−1^	26.3
Ca total^e^	g kg^−1^	248

^a^ISO 10390.

^b^ISO 23470.

^c^ISO 10694.

^d^Inorganic P extracted with water determined by the molybdenum blue method^45^.

^e^Acid digestion and ICP-ES analysis.

**Table 3:** Summary of two-way ANOVA analysis testing the effects of crop species, fertilization treatments, and their interaction on P-acquisition traits variations.

	Crop				Treatment				Crop × Treatment			
	DF	MSq	F	*p*	DF	MSq	F	*p*	DF	MSq	F	*p*
Acid phosphatase^a^	2	125.82	43.39	***	3	9.22	3.18	*	6	18.81	6.49	***
Alkaline phosphatase^a^	2	3.36	0.95	NS	3	12.29	3.46	*	6	4.72	1.33	NS
SRL^b^	2	8501.32	61.51	***	3	230.77	1.67	NS	6	521.40	3.77	**
Mn concentration^c^	2	6458.09	35.59	***	3	941.69	5.19	**	6	481.86	2.66	*
Carboxylates^d^	2	9480.40	7.05	**	3	651.67	0.49	NS	5	912.58	0.69	NS
pH^e^	2	0.02	4.90	*	3	0.00	0.43	NS	6	0.00	1.13	NS
AMF^f^	2	7.79	23.05	***	3	0.86	2.56	NS	6	0.96	2.83	*

Control soil samples were not included in the analysis. *p < 0.05, **p < 0.01, ***p < 0.001.

^a^Activity of the enzyme in the rhizosphere soil.

^b^Specific root length.

^c^Shoot Mn concentration.

^d^Sum of ^c^itrate, fumarate, malate, maleate, malonate exuded by roots the day of harvest.

^e^pH in the rhizosphere soil.

^f^Int^e^nsity of root colonization by arbuscular mycorrhizal fungi.

### Covariation of P-acquisition traits, shoot biomass and P concentration

**Figure 5:**
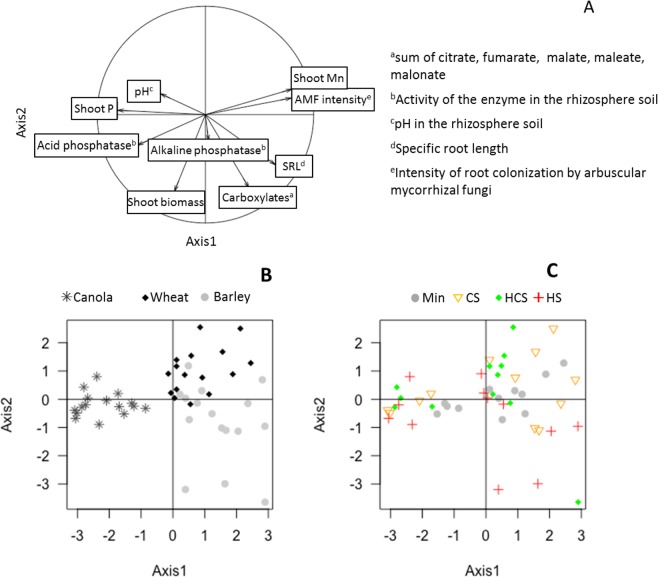
Variables projected in the plane determined by the first two principal axes (**A**) of a principal component analysis (PCA); positions of the points in the reduced space of the first two principal components after PCA grouped by crop (**B**) or fertilization treatment (Min: mineral P fertilizer, or fertilized with, CS: composted sludge, HCS: heated composted sludge, HS: heated sludge). (**C**) Control soil samples were not included in the analysis. Axes 1 and 2 accounted for 51% of the variance.

Figure 5 shows the results of the PCA performed on P-acquisition crop traits, shoot biomass and P concentration. The two principal components (PC) extracted by PCA accounted for 51% of the variance. The PC1 explained 39% of the variance and was negatively associated with shoot P concentration and acid phosphatase activity, and positively related to shoot Mn concentration and AMF intensity (Fig. 5A, Table S1). The PC2 explained 15% of the variance and was negatively correlated by shoot biomass, carboxylate exudates and specific root length (SRL).

Crops formed distinct groups along the PC1, wheat and barley points had positive values on PC1, while canola points had negative values (Fig. 5B). Barley and wheat points were spread on positive and negative values on PC2. When points were grouped by fertilization treatments, no clear patterns arose (Fig. 5C).

## Discussion

Efficiency of crop species to acquire P may depend on the type of P fertilizer applied. Previous work suggested that plant species have different P-acquisition traits, leading to preferential sources for P acquisition^17–19^. We hypothesized that the P-acquisition strategy of some crops could be more efficient in soil fertilized with organic waste, such as sewage sludge, than in soil fertilized with mineral P. The characterization of the relationships between the efficiency of P-acquisition strategies and type of fertilizer is a challenging step to optimize combinations of crop species or cultivars and P fertilizer type, thereby allowing more sustainable P fertilization practices.

The study of P-acquisition traits is needed to determine the different P-acquisition strategies of crops^12,25^. Strategies of P-acquisition can be highlighted by multivariate analysis of the main architectural, morphological, biotic and physiological root traits involved in P-acquisition in crop species^26^, i.e. the specific root length (SRL), leading to a greater volume of soil explored^13,27^, the release of acid phosphatases into the rhizosphere, hydrolyzing P-organic^16^; the release of protons, solubilizing P bound to Ca^14^; and the release of carboxylate, increasing Pi or P-organic desorption from clay minerals and Al and Fe oxides and hydroxides^28^, and the interactions with soil microbes, i.e. AMF root colonization and soil alkaline phosphatase activity. In our study, the PCA performed on P-acquisition traits, AMF colonization, alkaline phosphatase activity, shoot biomass and P concentration showed a clear separation by crop (Fig. 5B), indicating that the P-acquisition strategy depended on the nature of the crop. The shift in values along the PC1 clearly illustrates the contrasting P-acquisition strategy of canola compared with wheat and barley.

Canola was characterized by a high acid phosphatase activity in the rhizosphere, but a low SRL and a low carboxylate release (Fig. 5). The higher activity of acid phosphatase in the canola rhizosphere compared with that for wheat and barley (Fig. 4A) may result from a higher production by roots, and/or a higher stimulation of soil microbial activity that also produces acid phosphatase. However, a stimulation of microbial activity may also have increased the activity of alkaline phosphatase, produced by microbes only, while it was not observed here (Fig. 4B). In addition, a stimulation of microbial activity may have resulted from the release of carboxylate by roots, serving as substrate or signaling molecules to microbes^23^, while in our study canola was the crop releasing the lowest amount of carboxylate (Fig. 4E). These findings suggest that the higher acid phosphatase activity resulted from a greater release by roots of canola, rather than a stimulation of microbial activity. These results are in line with Wang *et al*.^29^, showing that canola produces more acid phosphatase than wheat and oat, and Tadano and Sakai^30^, who found that other Brassicaceae (e.g., cabbage and radish) are characterized by a high activity of rhizosheath acid phosphatase. Canola was the crop releasing the lowest total amount of carboxylate (Fig. 4E), but the amounts of malate and citrate released were similar to high amounts recorded in the literature for canola^31^ (Fig. S1B,C). Carboxylate excreted can induce P release by directly replacing P on ligand exchange surfaces, such as clays and Al or Fe (hydr)oxydes, which is the case of citrate, and/or by complexing metal ions bind to P^31^. In addition, previous studies suggested that carboxylate excreted can acidy the rhizosphere soil and in turn increase the dissolution of Ca-P^32^, but here the release of carboxylate was not correlated with any change of pH. The absence of acidification could be explained by the large buffer capacity of the calcium carbonate of the calcisol used in this study. The lower shoot Mn concentration of canola compared with that for wheat and barley (Fig. 4D) supports the lower carboxylate exudation observed (Fig. 4E). Our study suggests that the P-acquisition strategy of canola comprises a high acid phosphatase in its rhizosphere which promotes P-organic hydrolyze into readily available Pi.

Wheat and barley were characterized by a low rhizosheath acid phosphatase activity, but a high SRL and a high carboxylate release, especially for barley (Fig. 4). Among the carboxylate measured in our study, barley released principally malonate and malate, while wheat released principally malate and citrate (Fig. S1). Malate and citrate are both usually released by many crops in response to P deficiency^33^, however literature shows that wheat and barley release few amounts of carboxylate, except when they are Al-tolerant^31,34^, which is likely the case here. Carboxylates release by Al-tolerant plant can increase with Al concentration in soil^34^, but can also result from other toxicity. Christiansen-Weniger *et al*.^35^ showed that Al-tolerant wheat cultivar release more carboxylates than Al-sensitive, but suggested that the release was independent of Al stimulation. In our study, barley released also very large amounts of malonate but only in soils fertilized with sludges (Fig. S1A), which may result from a P deficiency or from another stress induced by sludge application, such as an increase of metals concentration in soil. However, this result was surprising, as malonate is not a carboxylate usually released by Graminaceous plants^33^. The identification of carboxylate was performed by HPLC like in many studies, but a mass spectroscopy analysis should be down to confirm that the peak we identified corresponded to malonate or another carboxylate. The higher SRL of wheat and barley compared with canola corresponds to thinner roots and a larger root system, for an equivalent root biomass, and can thus lead to a higher volume of soil explored. Wang *et al*.^36^ showed that barley had a greater total root length than canola with mineral P supply, but in their study this longer root system leaded to a higher P content for barley. In our study, the higher root length of wheat and barley did not lead to a higher P content than canola. This may result from the very low root colonization by AMF of wheat and barley, reaching a maximum of 3% in our study. Wang *et al*.^29^ showed wheat root colonization by AMF can reach 32 to 40%. Roots AMF colonization is traditionally promoted in nutrient-deficient soils^37^; in our study, Olsen-P in soil with sludges or mineral P application ranged from 10 to 12 mg kg^−1^ (Fig. 1) and can be considered as medium compared with data reported in the literature^38,39^. The low AMF colonization observed here most likely resulted from the lack of P deficiency in the fertilized soils, but can also resulted from the high soil pH, as pH is an important driver of fungal colonization of plants with AMF^40^. These results suggest that crop P-acquisition relies only partly on additional microbial enhancement. Our study suggests that the P-acquisition strategy of wheat and barley is to release carboxylate, that can increase P desorption from soil minerals^28^, and to develop long roots to increase the volume of soil explored.

Two contrasting P-acquisition strategies were determined: i) the strategy of crop species such as canola that stimulate the acid phosphatase activity, and ii) the strategy of crop species such as wheat or barley having a high SRL and a relatively high carboxylate release. These two strategies can have a different efficiency to take up P depending on the type of fertilizer applied, especially between the application of organic wastes adding organic P, such as sewage sludge, and the application of mineral P fertilizer. These different strategies can be used to optimize combination between the crop and the P fertilizer. For instance, crop species that stimulate the phosphatase activity might be more efficient to mineralize soil organic P, and thus to take up P in the presence of organic amendments, while crop species with a high release of carboxylate might be more efficient to acquire mineral P sorbed onto soil constituents, and thus to take up P in the presence of mineral P fertilizer. Nevertheless, optimizing combinations between fertilizer types and crop species should also be determined regarding the fertilizer impacts on plant-microbial interactions leading to additional crop P-acquisition^20^.

One of our hypothesis was that, in response to the supply of various forms of inorganic and P-organic, crop P-acquisition traits and interactions with microbial activity could have changed and increase the use of different P forms. Shahzad *et al*.^41^ have shown that organic amendments increased the amount of carboxylate in the rhizosphere of maize, while others studies have shown an increase of phosphatase activity in soil after sewage sludge application in incubation experiment^22^ or in the field^9,42,43^. These increases were mainly explained by stimulation of microbial activity induced by organic matter applied. Nevertheless, our results show that, irrespective of crop species, sludge application had little impact on P-acquisition traits, and on interactions with microorganisms (Table 3). Alkaline phosphatase activity and AMF colonization were not enhanced by application of sludge compared with mineral P and the intensity of AMF colonization remained very low with the application of any of the three sludges (Fig. S2). The present study suggests that organic matter applied with sewage sludge was not sufficient to promote additional crop P acquisition by stimulation of microbial activity and that P-organic added with sludge did not increase the production of acid phosphatase. Houben *et al*.^9^ found a higher microbial activity in soil after the application of similar sludges than tested here, but only when a high dose was applied; the dose of sludge applied here was thus probably too low to stimulate soil microbial activity. The present study shows that P-acquisition traits of the studied species did not change between sludge application and mineral P fertilizer application, suggesting that the form of P added had no influence on P-acquisition traits.

The P-acquisition strategy of each crop changed little with type of fertilizer applied, but each strategy can have a different efficiency to improve P uptake depending on the type of fertilizer applied. Some P-acquisition strategies can be more efficient to take up P from soil fertilized with organic waste, such as sewage sludge, than in soil fertilized with mineral P^17–19^. In our study, P-acquisition strategy of canola was particularly efficient to increase shoot P content from soil fertilized with sludge, as shoot P content of canola was higher in soil fertilized with any of the three sludges than with mineral P added at the same P dose (Fig. 3). Contrary to mineral P fertilizer, sludges contain P in organic forms (Table 1) and their application can thus increase the proportion of soil P-organic. As P-acquisition strategy of canola was to increase acid phosphatase activity in the rhizosphere, we can expect that the increase of acid phosphatase enhanced the mineralization of P-organic added with the sludge, which released Pi and, in turn, increased shoot P content of canola.

The contrasted efficiency of P-acquisition strategy of canola, wheat and barley can constitute a tool to optimize crop rotation with organic waste application. The present study shows that canola had the most efficient strategy to take up P from soil fertilized with sewage sludge, and potentially with other types of organic wastes. As sewage sludges slowly increase the plant-available soil P after their incorporation in soils^9^, canola should come first in the crop rotation, to improve crop P content from poorly-available P forms. Crop species such as wheat and barley that have a high SRL and a relatively high carboxylate release could be associated with soils having a high P-sorption capacity.

In conclusion, the P-acquisition strategy determined in soil fertilized with sewage sludge or mineral P was different for canola, and for wheat and barley. Wheat and barley had a higher specific root length and a higher root carboxylate release, and their shoot P content was equivalent with sludges or mineral P. By contrast, canola P content was higher with sludge, rather than mineral P was applied. This was attributed to its higher rhizosheath acid phosphatase activity which promoted mineralization of sludge-derived P-organic. Organic waste application should be associated with crop species such as canola with a high rhizosheath acid phosphatase activity in the rhizosphere.

One of the major challenges of P fertilization is to provide available P for plants while limiting the use of mineral P fertilizer. This study shows that the contrasting P-acquisition strategies of crops can be used to design efficient cropping systems with organic waste application.

## Materials and Methods

### Sewage sludges and soil

Three types of sewage sludge produced by the Parisian public sanitation service (Seine Aval wastewater treatment plant, SIAAP, Paris) were used: a composted sewage sludge (CS) treated by anaerobic digestion followed by composting with municipal green wastes; a heated sewage sludge (HS), treated by anaerobic digestion followed by a dewatering by thickening and thermal conditioning (heat exchange and heating during 45 min at 195 °C and 20 bars); and a heated and composted with municipal green wastes sludge (HCS). Sludge properties are given in Table 1. Phosphorus fractions were determined through the SMT (Standards, Measurements and Testing program) protocol^44^. Four P fractions in sludge were considered: Pi-water (soluble inorganic P), P-apatite (calcium bound P), Pi-non-apatite (Pi bound to aluminum (Al), iron (Fe) and manganese (Mn) oxides and hydroxides), and P-organic. The three sludges had an equivalent P-organic fraction, but the sludges that received a thermal treatment (HS and HCS) had a lower P-apatite fraction.

A calcisol (IUSS Working Group WRB, 2014) was sampled (5–20 cm) in Fouquenies, located in the North of France (49°28′N 2°02′E). Soil was air-dried and sieved at 2 mm before being analyzed for pH (ISO 10390), cation exchange capacity (CEC) (ISO 23470), organic C (ISO 10694), N-total (EN 13342), N-NH_4_, water-soluble Pi determined by the molybdenum blue method^45^, total concentration of P, Ca, Al and Fe (acid digestion and ICP-ES analysis) (Table 2) and metallic trace elements (Table S2). The calcisol used was a clay loam soil with a high concentration of Ca (258 g kg^−1^) and an initial low P availability (Pi-water < 1 mg kg^−1^).

### Greenhouse pot experiment

The three sludges were applied in pots containing 2.9 kg dry soil at the same P rate (0.06 g P kg^−1^), corresponding to an agronomic dose of 75 kg P ha^−1^. The mass of raw sludge applied correspondingly was 6 g kg^−1^ dry soil for CS, 1.8 g kg^−1^ dry soil for HS and 4.8 g kg^−1^ dry soil for HCS. Triple superphosphate (TSP) was applied at the same rate (0.06 g P kg^−1^) as a mineral control. Sludge and TSP were homogenized through the soil by thorough hand mixing. A control without fertilization was also included. The experiment was carried out in six replicates. Distilled water was applied to reach field capacity and a nutrient solution was applied during the growth, corresponding to a total application of 1.17 g kg^−1^ dry soil for N and 3.21 g kg^−1^ dry soil for K.

Wheat (*Triticum aestivum* L. var. *bergamo*), barley (*Hordeum vulgare* L. ‘Viva’) and canola (*Brassica napus* L. var. *astronom*) were sown separately and grown for three months in a greenhouse with the following climatic conditions: 16 h of light day^−1^, 24/16 °C (day/night). Eight seeds per pot of wheat and barley and four seeds of canola were sown. After one week, the plants were thinned to two plants per pot. A treatment without plant was also included. The experiment was carried out in six replicates. Distilled water was applied every two days during the growth period. After three months, aboveground parts and roots of plants were harvested separately. Rhizosheath soil, i.e. the soil adhering to the root surface within 2 mm after shaking, was collected and air dried.

Aerial parts of the plant were dried at 60 °C for 48 h, weighed and ground. A subsample was digested in 8 mL 65% nitric acid and 2 mL 37% hydrochloric acid in a microwave system (Mars 5, CEM Corporation, Charlotte, USA). Shoot P and Mn concentrations were determined by Inductively Coupled Plasma Mass Spectrometry (ICP-MS, Thermo Scientific XSERIES 2). Mature leaf Mn concentration is considered a proxy for rhizosheath carboxylate concentration^28,46^.

### Analyses of rhizosphere soil

#### Soil phosphorus availability

Soil phosphorus availability was assessed by anion exchange membrane^47^ and the Olsen methods^48^. The anion exchange membrane method involved mixing a mass of soil equivalent to 0.5 g dry soil with 60 mL of water and two anion exchange membranes (AEM) of 5 cm^2^ loaded with HCO_3_^−^ with an anion exchange capacity of 0.037 cmol_c_ cm^−2^ for 16 h. Then, AEM were removed from the soil-solution mixture, rinsed with ultra-pure water, and eluted for 16 h in 20 mL of 0.5 M HCl. The Olsen extraction was performed by shaking the soil with 0.5 M NaHCO3 at pH 8.5 (1:20 soil:liquid ratio) for 30 min. The concentration of P in each extract was then measured by ICP-MS (Thermo Scientific XSERIES 2).

#### Acid and alkaline phosphatase activities

Acid and alkaline phosphatase activities were assayed by the method of Tabatabai and Bremner^49^ which involves the determination of p-nitrophenol released by incubation at 37 °C for 1 h of 1 g soil with 0.2 ml toluene, 4 ml universal buffer (pH 6.5 for acid phosphatase and pH 11 for alkaline phosphatase) and 1 ml sodium p-nitrophenyl phosphate as substrate^50^.

#### Roots trait characterization

One plant per pot was used to collect carboxylate exudates. The day of harvest, after plants were removed from the soil and roots were cleaned with distilled water, the whole root system was dunked in 40 mL of 0.2 mM CaCl_2_ solution for 30 min. The solution was then frozen and lyophilized to concentrate carboxylate, 2 mL was centrifuged (2 min, 10,000 g) and 1.5 mL of supernatant solution was filtered through a sterile 0.22 µm millipore filter. Fifty microliters of filtrate were injected into the HPLC system (Thermo Fisher, San Jose, CA, USA) equipped with an auto-injector, a degasser, and a diode array detector. Malate, malonate, maleate, citrate and fumarate were separated according the method of Cawthray^51^ on a reverse phase Luna 5 µm C18 column (250 mm 4 mm (phenomenex)) at 25 °C by using an isocratic mobile phase: 25 mM KH_2_PO_4_: methanol (99:1; v-v) buffered at pH 2.40. The flow rate was set at 1 mL min^−1^. The UV signal was recorded at 210 nm.

The specific root length (SRL) was determined on the same plant used for exudate analysis. After collecting carboxylate exudates, roots were separated from the aerial part and total root length was determined using a scanner (Calibrated Color Optical scanner STD4800 with special lighting system. S/N URUW009925-6714112, Optical Resolution 4800 dpi, max. scan area: 22 × 30 cm) and WinRHIZO (Regent Instruments Inc., Quebec, Canada) software. The entire root was then dried at 60 °C for 48 h and weighed. The SRL was calculated as the ratio between total root length and root dry biomass.

The second plant of the pot was used to determine the intensity of arbuscular mycorrhizal fungi (AMF) root colonization for wheat and barley only. As most species belonging to Brassicaceae, canola cannot be colonized by AMF^18^. A root subsample of the plant was cleared in 10% (w/v) KOH, stained with trypan blue^52^, and 30 random sections of 1 cm were observed under a microscope (100x enlarged) to determine the percentage of root surface colonized by AMF. Six categories were used, 0: 0%, 1: 0–1%, 2: 1–10%, 3: 10–50%, 4: 50–80%, and 5: >80%. The intensity of AMF colonization of each plant was calculated as follow^53^:

$$AMF\,intensity\,( \% )=\frac{95n5+70n4+30n3+5n2+n1}{number\,of\,sections\,colonized}$$

with ni the number of sections belonging to the category i.

### Data processing and analysis

Data were statistically analyzed with the R package^54^. A Kruskal-Wallis test followed by a Dunn test of multiple comparisons using ranked sums revealed significant differences (*p* ≤ 0.05) among fertilization treatments or crop species. Two-way ANOVAs were performed to test the effects of crop species, the fertilization treatment and their interaction on the variations of each P-acquisition trait.

To examine the covariation of P-acquisition traits and P content regarding the crop or the fertilization treatment, a principal components analysis (PCA) was performed on the following variables: shoot P concentration (shoot P), shoot biomass, specific root length (SRL), intensity of AMF colonization (AMF intensity), total amount of carboxylate in rhizosheaths (carboxylate), shoot Mn concentration (shoot Mn), acid and alkaline phosphatase activities in the rhizosheath, and pH in the rhizosheath. The variables were submitted to PCA; rotated orthogonal components were extracted, and the relative scores were determined. Control soil samples were not included in the PCA in order to compare only soils that received the same amount of P.

## Supplementary information

Supplement file

## Supplementary information

is avilable for this paper at 10.1038/s41598-019-51204-x.
