# Supplementary material for: Phosphorus-acquisition strategies of canola, wheat and barley in soil amended with sewage sludges
Source: Sci Rep. 2019 Oct 16;9:14878. doi: 10.1038/s41598-019-51204-x (PMC6795825; doi:10.1038/s41598-019-51204-x)

Phosphorus-acquisition strategies of canola, wheat and barley in soil amended with sewage sludges

C. Nobile, D. Houben, E. Michel, S. Firmin, H. Lambers, E. Kandeler, M-P. Faucon

Table S1: Variables contributing to the two principal components (PC) of the principal components analysis. Contributions are printed in percentage and the sign is the sign of the coordinates.

	PC1	PC2
Shoot P concentration	**-66**	0
Shoot biomass	-8	**-50**
Shoot Mn concentration	**64**	6
SRL[a]	42	**-21**
Carboxylate exudates[b]	16	**-46**
AMF intensity[c]	**64**	2
pH[d]	**-17**	4
Acid phosphatase activity[e]	**-40**	-8
Alkaline phosphatase activity[e]	0	-6

[a]Specific root length

[b]Sum of citrate, fumarate, malate, maleate, malonate released by roots on the day of harvest

[c]Intensity of root colonization by arbuscular mycorrhizal fungi

[d]pH in the rhizosphere soil

[e]Activity of the enzyme in the rhizosphere soil

Table S2: Metallic trace elements in sludges (Aqua regia extraction: EN 13650; Elements determination: EN ISO 11885). CS: composted sludge; HS: heated sludge; HCS: heated composted sludge.

	Unit, dry mass	**CS**	**HS**	**HCS**
Cr		54.2	61.7	38.6
Cu		217	781.2	378
Ni		24.3	26.2	21.0
Zn	mg kg^{-1}	483	1773.3	965
Cd		1.9	3.4	2.4
Pb		53.8	138	85.8
Hg		0.2	1.711	0.9

Figure S1: Amounts of carboxylate released by wheat, canola and barley grown for three months in a calcisol fertilized with mineral phosphorus (P) (Min), or with sewage sludge (CS: composted sludge, HCS: heated composted sludge, or HS: heated sludge). Error bars are standard errors (*n* = 4). NA is not available. Different letters indicate significant difference among crops at the 0.05 level; ns is not significant.

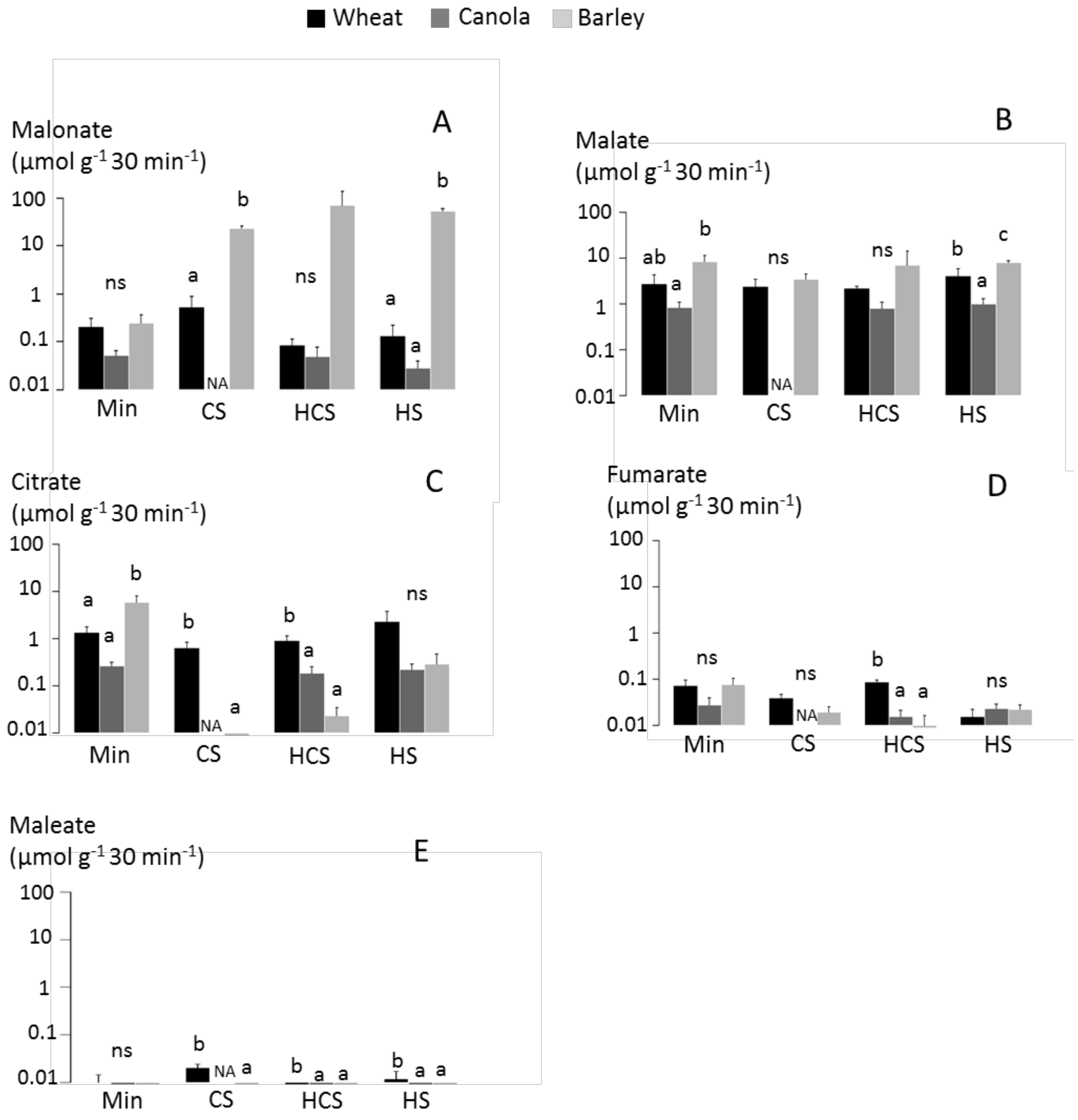

Figure S2: Traits involved in phosphorus (P) acquisition for wheat, canola and barley after three months of growth in greenhouse on a calcisol fertilized with mineral P (Min), or sewage sludge (CS: composted sludge, HCS: heated composted sludge, or HS: heated sludge). Error bars are standard errors (n = 4). Different small and big letters indicate significant difference at the 0.05 level among fertilization treatments and among crops, respectively; ns and NS is not significant.

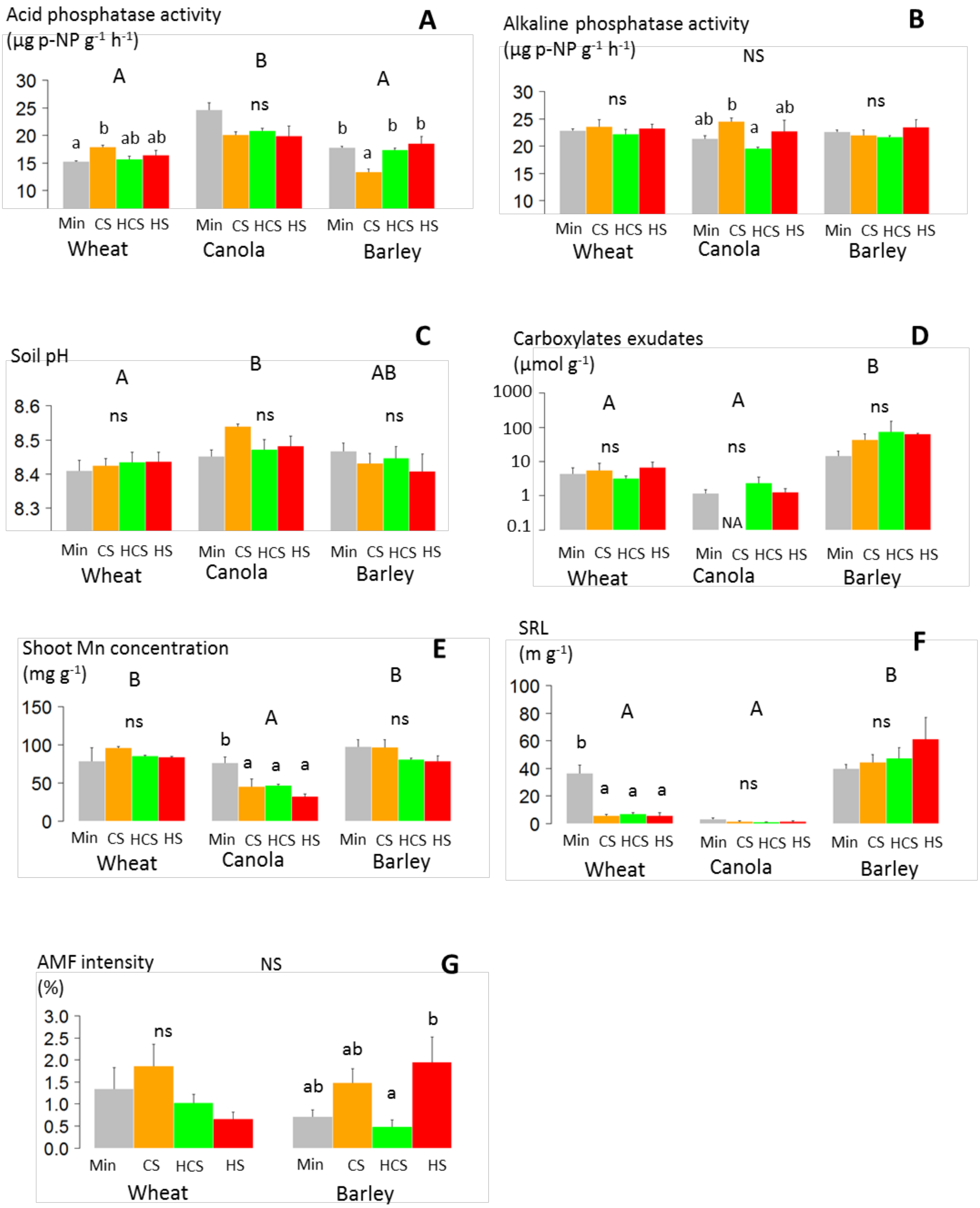

Supplement: Supplementary file 1 — Supplement file [file 41598_2019_51204_MOESM1_ESM.pdf]
